# Histone Modifications, Modifiers and Readers in Melanoma Resistance to Targeted and Immune Therapy

**DOI:** 10.3390/cancers7040870

**Published:** 2015-09-25

**Authors:** Stuart J Gallagher, Jessamy C Tiffen, Peter Hersey

**Affiliations:** 1 Melanoma Immunology and Oncology Group, Centenary Institute, University of Sydney, Camperdown 2050, Australia; j.tiffen@centenary.org.au (J.C.T.); peter.hersey@sydney.edu.au (P.H.); 2 Melanoma Institute Australia, Crow’s Nest 2065, Sydney, Australia

**Keywords:** BRAF, RAF, MEK, immunotherapy, HDAC, histones, methyltransferase, bromodomain, BET, chromatin modifiers, resistance, PRC2, EZH2, PD-L1, PD-1

## Abstract

The treatment of melanoma has been revolutionized by new therapies targeting MAPK signaling or the immune system. Unfortunately these therapies are hindered by either primary resistance or the development of acquired resistance. Resistance mechanisms involving somatic mutations in genes associated with resistance have been identified in some cases of melanoma, however, the cause of resistance remains largely unexplained in other cases. The importance of epigenetic factors targeting histones and histone modifiers in driving the behavior of melanoma is only starting to be unraveled and provides significant opportunity to combat the problems of therapy resistance. There is also an increasing ability to target these epigenetic changes with new drugs that inhibit these modifications to either prevent or overcome resistance to both MAPK inhibitors and immunotherapy. This review focuses on changes in histones, histone reader proteins and histone positioning, which can mediate resistance to new therapeutics and that can be targeted for future therapies.

## 1. Melanoma Therapy with MAPK and Immunotherapy

Historically, unresectable metastatic melanoma was almost impossible to treat because of its resistance to most chemotherapy and radiotherapy [1]. This has changed with the advent of two new types of therapies for melanoma—mitogen-activated protein kinase (MAPK) inhibitors and immunotherapy. Approximately 40% of cutaneous melanoma patients have tumors with an activating mutation in the BRAF gene (BRAF V600E/K), which activates the MAPK pathway via a phosphorylation cascade that activates MEK and ERK [2]. Phosphorylated ERK has a number of targets including transcription factors ELK and ETS that lead to enhanced cell proliferation and survival signals. Patients with tumors harboring BRAF mutations are now treated with inhibitors of mutant BRAF (vemurafenib or dabrafenib), increasingly in combination with MEK inhibitors such as trametinib or cobimetinib [3,4]. Although initial response to these therapies is high (~80%), almost all patients relapse with tumors due to acquired resistance to these drugs [5,6,7]. Primary resistance in the 20% of patients that do not show an initial response is also a problem.

Immunotherapy has also provided breakthroughs in melanoma treatment by overcoming immune evasion mechanisms frequently used by melanoma cells. Cutaneous melanoma has the highest mutation rate of all cancers, which in theory should provide plenty of neo-antigens to attract immune surveillance and T-cell mediated killing [8]. However melanoma evades the immune system by expressing Programmed Cell Death Ligand 1 (PD-L1), which binds to the Program Cell Death 1 (PD-1) receptor on T-cells and inhibits their cytotoxic T-cell activity. PD-1 inhibitors (pembrolizumab and nivolumab) and another immune checkpoint inhibitor against CTLA4 (ipilimumab) provide more durable responses than MAPK inhibitors but are also plagued by primary and acquired resistance [9]. Response rates to immunotherapy are lower than to MAPK inhibitors—10%–20% for ipilimumab, 30%–40% for PD-1 inhibitors and 50%–60% for combination of ipilimumab and anti-PD-1 [10,11,12,13]. The superiority of combined PD-1/CTLA4 therapy over single drug treatment was shown in a recent randomized trial in which the progression free survival (PFS) was 11.5 months for the combination compared to 6.9 months for Nivolumab alone or 2.9 months for ipilimumab alone [11,12,13,14,15,16,17].

Despite these encouraging successes, acquired and intrinsic resistance to MAPK inhibitors and immunotherapy are the most important challenges facing melanoma therapy today. Concerted efforts of researchers worldwide have found a number of different gene mutations or amplifications that cause resistance to MAPK inhibitors, explaining resistance in about half of patients failing MAPK inhibitors. Resistance to immunotherapy has been associated with low numbers of tumor infiltrating lymphocytes (TILs) [18] but the mechanisms involved remain poorly understood.

## 2. Melanoma Resistance to MAPK Inhibitors and Immunotherapy

Diverse mechanisms cause resistance against MAPK treatment in melanoma [19,20]. Mechanisms of resistance involve either reactivation of MAPK-pathway signaling or activation of other survival and growth pathways, allowing the melanoma cells to proliferate despite MAPK inhibition. Alternatively, the cell state of the melanoma isn’t entirely dependent on MAPK signaling, allowing cell survival and growth even when MAPK signaling is blocked. These mechanisms will be discussed more fully in the subsequent sections, but can be divided into a number of classes.

- Resistance caused by strong anti-apoptotic signaling
- Mutations that reactivate the MAPK pathway
- Mutations that activate alternative survival pathways
- Receptor tyrosine kinase activation/upregulation to provide alternate survival signals
- Presence and selection of slow cycling, drug resistant melanoma cells
- MITF, cAMP and NF-κB related mechanisms

Resistance of melanoma to immunotherapy is less well defined. Primary resistance is the largest problem—even with combined anti-PD-1/anti-CTLA4 treatment, progressive disease is the best response in almost 30% of patients [11]. Both the biology of the immune system and melanoma cells needs to be addressed when looking to modulate immunotherapy—from antigen presentation, expression of immune checkpoints on melanoma and immune cells, and the activity, type and number of immune cells. Epigenetic histone modifiers have been shown to modulate both immunogenicity of melanoma cells and immune function but often in an opposing fashion. This review investigates the contribution of histone changes and histone modifiers in causing resistance and how they can be leveraged to overcome or prevent resistance.

## 3. Epigenetic Changes—A Brief Overview

Epigenetic regulation refers to (mitotically) heritable changes in gene expression that are not directly the result of changes in DNA sequence. This review focuses on covalent modifications of histones, histone modifiers and histone-binding proteins. DNA methylation and non-coding RNAs are also important epigenetic events and are reviewed elsewhere [21,22,23].

Histone modifications provide epigenetic control of gene expression and are viewed as being more dynamic than DNA methylation marks, which, with the exception of TET-meditated changes, are considered to be more permanent [24]. Different chemical groups can be added to histones by proteins broadly classified as histone writer proteins [25,26]. The best studied of these modifying “marks” is acetylation and methylation. These groups can be removed by histone “eraser” proteins. Finally, modifications on histones act as a template, allowing binding by modification-sensitive histone reader proteins. The reader proteins recruit transcription factors or repressors to modulate gene expression [27]. Finally, nucleosome structure and the exact histone variants that make up a nucleosome affect gene transcription. A brief summary of these main epigenetic factors and their context in melanoma biology is given below.

### 3.1. Histone Acetylation

Histones can be acetylated at lysine (K) residues by the transfer of acetyl groups from acetyl-CoA to the ε-amino group at the terminal of the lysine side chain [28]. The transfer of the acetyl groups is catalyzed by histone acetyl transferases (HATs) and the groups can be removed by histone deacetylases (HDACs), with the balance of these opposing factors determining the level and state of histone acetylation. The addition of the acetyl group neutralizes the lysine’s positive charge, thereby weakening the interaction with the negative charge of DNA, leading to a more open chromatin structure allowing easier access to transcription factors and increased transcription. Lysine residues on the tails of H3 and H4 are the best studied targets of histone acetylation but other more internal residues (such as H3K56) may also be acetylated. Histone acetylation also influences transcription by acting as a binding target for histone reader proteins. Finally, acetyl groups on histones must be removed to allow the deposition of other histone marks, such as the transcriptionally permissive H3K4me3 or repressive H3K27me3, thereby adding another layer of transcriptional control.

Changes in histone acetylation have been noted for many genes during melanomagenesis and may contribute to the downregulation of specific tumor suppressor genes such as p14ARF and p16INK4a [29,30], thus factors modulating histone acetylation have been the subject of much study. HDACs have commanded the most attention due to the increasing number of inhibitors available for these proteins. HDACs are divided into four classes including class I (HDAC 1, 2, 3 and 8), which are predominantly nuclear, class II HDAC (HDAC 4, 5, 6, 7, 9 and 10) that shuttle between the nucleus and cytoplasm, class III that consist of NAD dependent sirtuins, and class IV (HDAC11) [31,32,33]. HDAC expression is generally elevated in cancer for example HDAC1, 2, 3 and 6 are reported to be upregulated in various cancers [31] but some HDACs, such as HDAC1, 2 and SIRT1, may also be lost or decreased [34,35]. Relatively little is known about HDAC expression in melanoma. HDAC8 expression is associated with improved survival in melanoma, but HDAC1 and HDAC8 also correlate with increase phosphorylated p65—a subunit of the NF-κB complex, which is associated with resistance to MAPK inhibitors [36,37].

Much of the work studying HDAC function has been performed using small molecule inhibitors and the specificity of these inhibitors varies [31]. Additionally, HDACs and HATs have histone-independent targets, which can have significant cellular effects, thus, one must be cautious when comparing the results of studies using different HDAC inhibitors. Histone-independent targets of HDAC proteins include HSP90, p53 and NF-κB subunit p65. Hyperacetylation of chaperone protein HSP90 following HDAC inhibition leads to degradation of signaling molecules c-RAF and Akt—both important mediators of growth and MAPK inhibitor resistance in melanoma. Additionally, HSP90 hyperacetylation causes degradation of the upstream receptor tyrosine kinases ERBB1 and ERBB2 which can promote MAPK inhibitor resistance. Alteration of p53 activity by HDAC inhibitor driven hyperacetylation may alter melanoma resistance to therapy [38,39,40] and NF-κB activation may alter cytokine production, anti-apoptotic protein transcription and immune response [41,42,43]. More extensive summaries of histone-independent HDAC targets can be found elsewhere [31,44,45].

### 3.2. Histone Methylation

Histones are methylated by histone methyltransferases on lysine or arginine (R) amino acids, often leading to repressive chromatin states (e.g., methylation of H3R2, H3R8, H3K9, H3K27). A number of histone methyltransferases are implicated as having a role in melanoma, especially enhancer of zeste homolog 2 (EZH2)—the catalytic subunit of the Polycomb Repressive Complex 2 (PRC2) complex which represses transcription by adding the H3K27me3 mark [46]. Subsequent histone ubiquitination by the PRC1 complex leads to deep transcriptional repression. EZH2 can also associate with DNA methylases to link histone methylation with DNA methylation [46]. Activating mutations in EZH2 occur in 3% of melanoma, where it functions as a driver of melanoma progression, and its expression may be upregulated in a further cohort of melanoma [47,48,49,50]. EZH2 function can be inhibited by a number of small molecules and EZH2 inhibition in melanoma reduces cell growth and metastases [48,50]. Not all histone methylation marks are repressive and H3K4 methylation is a marker of active transcription from a gene.

### 3.3. Histone Readers

Histone modifications not only strengthen or weaken the interaction between DNA and histones, but also serve to allow the recruitment of epigenetic regulators, which bind specific covalent modifications or patterns of modifications on histones [51]. Reader proteins have a variety of different domains which bind to specific alterations—acetylated lysine residues are bound by bromodomains and plant homology domains (PHD), methylated lysines are bound by HD, chromo, WD40, Tudor, double/tandem Tudor, MBT, Ankyrin Repeats, Zf-CW and PWWP domain proteins. Phosphorylated serine residues of histones are bound by BRCT domain containing MDC1 and by 14-3-3 family (see [27] for full review). Histone reader proteins can alter transcription by recruiting other enzymes, such as the positive transcription elongation factor (P-TEFb) or cause further histone modifications and many histone readers also have catalytic ability [27,51]. The bromodomain and extra terminal (BET) family of histone readers bind acetylated histones and have attracted some attention in melanoma. BET family members BRD2 and BRD4 are over expressed in melanoma and are able to be inhibited by a number of relatively novel drugs such as JQ-1 and I-BET151, leading to reduced melanoma growth, cell death and reduction in NF-κB activity [52,53,54,55]. The PhD domain containing ING1 protein has also been reported to be over expressed in melanoma [56].

### 3.4. Chromatin Structure (SWI/SNF)

While 146 nucleotides wrap around a histone to form a nucleosome, there is a variable number of nucleotides between each nucleosome. These more exposed nucleotides, which may typically number 50–100, are more accessible to transcription factors and therefore nucleosome spacing and location is an important determinant of transcription. The SWI/SNF chromatin remodeling complex can determine nucleosome location. It is comprised of an ATPase subunit (either BRG1 or BRM) and 8–14 BAF members. Inactivating mutations in this complex (ARID2A, ARID1A and SMARCA4) are found in 13% of melanomas [47,57], highlighting their role as tumor suppressors and emphasizing the importance of this complex in normal biology and disease. Ovarian cancer cell lines that harbor an inactivating mutation in ARID1A were recently found to be dependent on PRC2 and thus sensitive to EZH2 inhibition [58]. At least one of the two core ATPases in SWI/SNF (BRG1 or BRM) is required for melanoma growth, and they may regulate different sets of target genes [59]. ATRX, another SWI/SNF chromatin remodeler has also been shown to be reduced with melanoma progression [60].

### 3.5. Histone Variants

The histone core around which DNA is wrapped typically contains two of each of the canonical histones—H2A, H2B, H3, and H4 [61]. However canonical histones can be replaced by variant histones which have a different sequence and properties. Variants of H2A and H3 are most common and are inserted into specific genomic areas by histone chaperones, resulting in altered chromatin structure, modifications, and gene transcription [61]. Histone variant H2A.Z is increased in melanoma and other cancers [62] and consists of two sub-forms (H2A.Z.1 and H2A.Z.2) that only differ by three amino acids but are transcribed from distinct gene loci. H2A.Z.2 is highly expressed in melanoma and is bound by and stabilizes histone reader protein BRD2, leading to activation of genes, especially E2F targets that promote cell cycle progression [53]. H2A.Z.2 deficiency increases sensitivity to MEK inhibitor treatment and the involvement of H2A.Z.2 in causing resistance to MAPK inhibitors or immunotherapy in patients remains to be explored [53,63]. Histone 3 variant H3.3 is also associated with E2F target gene expression and overexpression of H3.3 leads to repression of E2F target genes and senescence [64]. Histone variant macroH2A suppresses melanoma progression via suppression of CDK8 expression and expression of macroH2A is generally lost with melanoma progression [62]. Targeting of these variants or the chaperones that deposit them into specific regions of chromatin could alter the sensitivity of melanoma cells to MAPK inhibitors or immunotherapy.

## 4. Overcoming Resistance to MAPK Inhibitors by Epigenetic Modulators

### 4.1. Reducing Intrinsic Anti-Apoptotic Signaling in Melanoma

Melanoma cells are intrinsically resistant to apoptosis which confers resistance to a variety of cytotoxic insults—from DNA damaging drugs, radiation and a variety of chemotherapeutic agents [65]. There are many pathways to cell death [66], but a central pathway is the caspase driven apoptosis pathway, which is mediated in-large part by the Bcl-2 family of proteins which control depolarization of the mitochondria [67,68]. Mitochondria are depolarized by Bax and Bak which are controlled by the balance of other pro or anti-apoptotic members of the Bcl-2 family [69]. Once depolarized, the contents of the mitochondria are released which activates caspases or caspase-independent molecules which complete the induction of cell death [65,70]. In the balancing act of pro- *vs*. anti-apoptotic proteins, melanoma cells have the dial turned to survival [65,71,72,73]. High levels of MAPK and/or PI3K signaling typical in melanoma increase levels of anti-apoptotic Bcl-2, Mcl-1, Bcl-XL, survivin and XIAP [65,74,75,76,77,78,79] while suppressing expression of the potent apoptotic inducer Bim and sequestering pro-apoptotic Bmf to the cytoskeleton [75,78,80,81,82,83].

**Figure 1:**
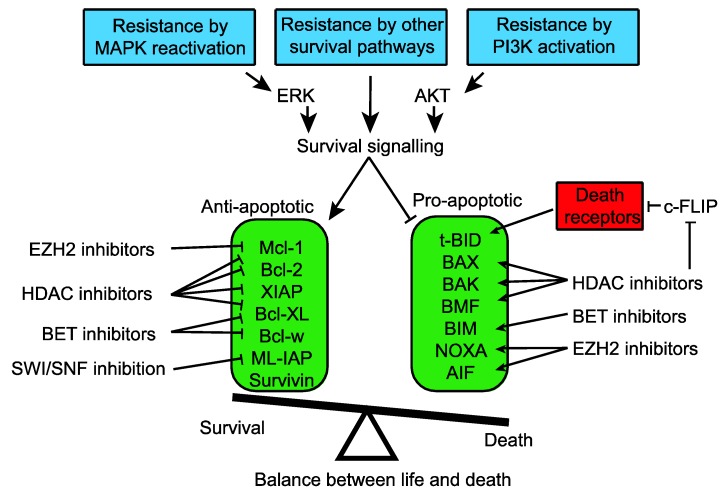
Mechanisms conferring resistance to MAPK inhibitors restore survival signaling by decreasing pro-apoptotic proteins and increasing anti-apoptotic signals. A variety of epigenetic modulators can push the balance back to signaling that favors cell death.

MAPK inhibitors cause apoptosis by adjusting this balance of proteins, most notably by inducing BIM, Bmf and reducing Mcl-1 [82,84,85]. This shift towards an apoptotic state is partly offset by a decrease in NOXA [86] but is often enough to trigger apoptosis. Melanoma cells which have high enough levels anti-apoptotic proteins can resist death in response to MAPK inhibitors and instead the primary response is cell cycle arrest. Epigenetic modifiers—especially HDAC inhibitors, can play a key role in switching the balance to an apoptotic state (Figure 1).

#### 4.1.1. Histone Acetylation, HDAC Inhibitors and Apoptotic Proteins in Melanoma

Inhibitors of HDACs create a pro-apoptotic environment that increases the cell death in response to MAPK inhibitors and can also cause cell death alone [87,88,89]. HDAC inhibitors increase or decrease the expression of hundreds to thousands of genes [90,91] and many of the gene changes shift the balance towards apoptosis. In melanoma, HDAC inhibitors inhibit expression of anti-apoptotic proteins survivin, Bcl-XL, Bcl-2, Mcl-1, XIAP, mostly via decreased transcription [87,92,93,94,95,96]. HDAC inhibitors differ in their ability to suppress individual anti-apoptotic proteins—for example suberanilohydroxamic acid (SAHA/Vorinostat) suppressed Bcl-XL levels in melanoma cells while valproic acid (VPA) did not [94]. These differences are likely due to differences in the HDACs targeted by different inhibitors—SAHA has a broader inhibitory effect on HDAC than VPA [31].

Pro-apoptotic proteins are also induced by HDAC inhibitors, for example suberohydroxamic acid (SBHA) induced BIM, BAX and BAK in melanoma cells [87]. Bax can be upregulated by the HDAC inhibitor sodium butyrate, although this was p53 dependent and may be a result of p53 acetylation, rather than direct changes in histone acetylation [97]. Cell cycle inhibitor and p53 target p21 (CDKN1A) is frequently reported to be upregulated by HDAC inhibitor treatment in a p53 independent fashion, which is the result of direct action of increased histone acetylation, chromatin remodeling and RNApol II recruitment to the CDKN1A promoter [98].

The extrinsic pathway of apoptosis, which activates caspases independent of mitochondria, can also be targeted by HDAC inhibitors which reduce levels of c-FLIP—an inhibitor of TNF-related apoptosis inducing ligand (TRAIL)—the class I HDAC inhibitor MS-275 rendered melanoma more sensitive to TRAIL killing [92,99].

#### 4.1.2. Histone Methylation

Inhibition of the histone methyltransferase and PRC2 member EZH2 leads to melanoma cell death by induction of AIF (apoptosis inducing factor) release from mitochondria [48]. Caspase independent apoptosis was also associated with a decrease in Mc1-1 and an increase Noxa, although the events preceding mitochondrial depolarization remain unclear. As persistently high Mcl-1 levels and decreased NOXA impede MAPK inhibitor-induced cell death, the combination of EZH2 and MAPK inhibition is rational [70].

#### 4.1.3. Histone Readers

The Bromodomain and Extra Terminal (BET) family of histone reader proteins also push melanoma cells towards apoptosis. BET proteins bind acetylated lysines on histone H3 and H4 and recruit RNA polymerase II to drive transcription and two BET proteins—BRD2 and BRD4 are increased during melanoma progression [52,53]. Inhibition of BET proteins leads to an increase in BIM, which may cooperate with BIM induction following MAPK inhibition and early data suggest that combining BET inhibitors and MAPK inhibition may be advantageous in melanoma [52,55,100]. Combining HDAC with BET further enhances apoptosis in melanoma and could be an alternate strategy [95].

#### 4.1.4. SWI/SNF

SWI/SNF can promote melanoma survival by remodeling the IAP promoter leading to enhanced MITF-driven BIRC7 (Livin/ML-IAP) expression [101].

### 4.2. Mutations that Re-Activate the MAPK Signaling Pathway

**Figure 2:**
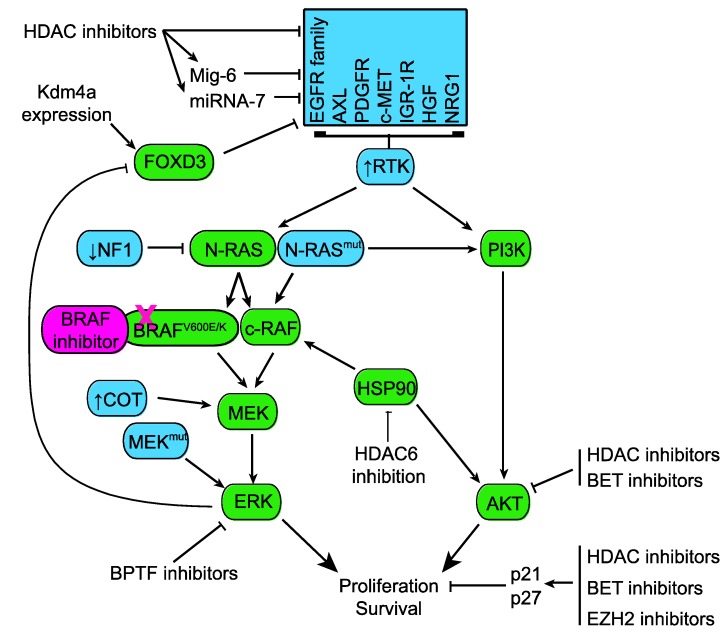
Melanoma cells develop resistance to MAPK inhibitors by mutating (mut) or altering expression of a number of proteins which reactivate MAPK signaling and/or activate alternative survival pathways such as the PI3K pathway. Some alterations known to drive resistance are shown in blue. Targeting epigenetic regulators may be able to overcome these resistance mechanisms by targeting the resistance mechanism directly or downstream signaling.

Many of the described mechanisms causing BRAF inhibitor resistance are mutations that reactivate the MAPK signaling pathway—for review see [19,20,102,103]. These may be mutations activating upstream components (NRAS Q61K/R or inactivation of NF-1), activating mutations in downstream targets such (MEK mutation or COT upregulation) and BRAF amplifications or BRAF truncation and splice mutants that are resistant to inhibition [104]. Epigenetic modifiers may serve to overcome some of these resistance mechanisms as well (Figure 2).

Not all mutations that cause resistance are created equal. For example a number of mutations in MEK I or II have been reported [19], and while some clearly provide strong BRAF-independent activation, others seem to be only weakly activating. Likewise, BRAF amplifications may not be completely protective, but may provide just enough survival signaling to allow a tumor to escape therapy. This “just enough” signaling model is supported by work showing that weak upstream MAPK pathway activation is sufficient to cause resistance to BRAF inhibitors [105]. In these situations, combination with HDAC inhibitors may be advantageous, as the HDAC inhibitor would increase death signaling while also assist to block cell cycle through p21 induction, as described in the previous section.

Upregulation of CRAF or reactivation of MAPK signaling via CRAF may be attenuated by the HDAC inhibitor LAQ826. LAQ826 inhibits HDAC6 activity to increase acetylation of HSP90, leading to a reduction in CRAF levels [106]. Another HSP90 client protein, AKT is also reduced by this treatment, which would be beneficial in reducing survival signals mediated by the PI3K pathway.

Blocking ERK signaling may be possible via targeting of the chromatin remodeler BPTF. BPTF has recently been reported to confer resistance to MAPK inhibitors and suppressing BPTF expression lead to a decrease in transcription of ERK, reduced total- and phospho-ERK protein levels and resulted in an increase in sensitivity to BRAF inhibitors [107].

### 4.3. Mutations that Activate Alternative Survival Pathways

The PI3K pathway is frequently activated in MAPK-inhibitor resistant melanomas to provide survival and growth signals [108]. HDAC inhibitors can blunt the signaling of the AKT pathway in a number of ways. Reduction of the AKT-inhibitor phosphatidylinositol 4,5-bisphosphate-5-phosphatase by histone hypoacetylation is mediated by HDAC2 and HDAC3 and can be reversed by HDAC inhibitor treatment [109]. HDAC inhibitors also prevent HDAC deacetylation of HSP90, leading to reduced HSP90 chaperone activity and a decrease in HSP90 target AKT3 [106]. Combined inhibition of HDAC and BET reader proteins also lead to a potent reduction in AKT phosphorylation that was related to a decrease in activation of YAP [95]. As discussed in the next section, HDAC inhibitors can also reduce RTK signaling, which reduces activation of AKT and other pathways. Dual PI3K/HDAC inhibitors have been designed and may offer benefits of tighter PI3K control and concomitant HDAC inhibition [110,111].

### 4.4. Receptor Tyrosine Kinases Driving Resistance

Upregulation or activation of receptor tyrosine kinases (RTKs) is one resistance strategy used by melanoma cells. RTK signaling activates either the PI3K signaling pathway, to provide alternative growth signals to overcome MAPK inhibition, or reactivates the MAPK pathway by signaling though CRAF—a pathway that can be blocked to some extent by MEK inhibition, explaining in part the advantages seen with concurrent BRAF and MEK inhibition. Upregulation of various RTKs or their ligands have been reported in MAPK-inhibitor resistant melanoma, including PDGFRβ, IGF-R1, HGF, FGFR2, NRG1 and the EGFR family (EGFR, ERBB2, ERBB3, ERBB4) [112,113,114,115,116]. While melanoma cell populations that express high levels of RTK may be selected and expand following MAPK inhibitor treatment, FOXD3 is increased by BRAF inhibitor treatment and transcriptionally induces ERBB3 expression, providing an early adaptive resistance response in melanoma [117,118,119]. FOXD3 expression is epigenetically controlled during development by histone and DNA methylation and inhibition of the activating H3K9me3 mark by overexpression of histone lysine demethylase Kdm4a can reduce its expression [120].

Multiple RTKs can be co-expressed in resistant melanoma cells, with expression clusters involving EGFR, ERBB3, AXL and PDGFR being identified [37,121,122] leading to multiple resistance drivers that are difficult to target with single drugs. The RTK driven resistance is often difficult to overcome with RTK inhibitors, which vary in specificity and effect between different RTKs [121].

HDAC inhibitors could play a role in treating these melanomas with RTK driven resistance. HDAC inhibitors have been reported to downregulate a variety of RTK including EGFR, ERBB2, ERBB3, c-MET, IGF-1R [123,124,125,126,127,128], or induce EGFR inhibitor MIG-6 [129]. The effects of HDAC inhibitors on RTK suppression are dependent on the HDAC inhibitor used—trichostatin A (TSA) reduced EGFR expression by up-regulating miRNA-7 but SAHA did not have this affect [130]. Expression of any of HDAC1, 2 or 3 was sufficient to increase EGFR expression in colorectal cells, and in a similar fashion specific reduction of any of these individual HDAC proteins by shRNA could decrease EGFR expression, although not as potently as pan-HDAC inhibition by TSA or SAHA [124]. The inhibition of EGFR was caused by reduced histone acetylation and SP1 recruitment to the EGRF promoter [124].

### 4.5. Slow-Cycling, Drug Resistant Melanoma

Epigenetic modifiers may also benefit MAPK inhibitor treatment by killing off slowly cycling melanoma cells which are resistant to BRAF/MEK inhibition. Drug resistant populations of cells have been identified as having high levels of JARID1A, a histone demethylase that associates with HDAC and is associated with elevated IGF-1R expression and resistance to RAF inhibition. Although JARID1A is a histone demethylase, its association with HDAC means that it is affected by HDAC inhibitor treatment, and HDAC treatment increased death in response to the AZ628 RAF inhibitor as well as the emergence of resistant cells. The existence of these slow growing, drug-resistant side populations, which have also been reported to have expression of JARID1B has been reported by others [131,132,133,134] and might be effectively targeted by HDAC inhibitors, which also kill non-proliferating cells [135].

### 4.6. MITF Related Resistance Mechanisms

MITF is the master melanocytic regulator, a transcription factor that drives the expression of a suite of genes necessary for the formation of melanocytes and melanogenesis. Expression of MITF in melanoma is variable and is driven by a number of factors including LEF/β-catenin and SOX10 in conjunction with CREB (cyclic AMP response element binding protein), a cyclic AMP responsive cofactor [136,137,138].

#### 4.6.1. The MITF/NF-κB/AXL Axis

Melanomas with low levels of MITF are resistant to MAPK inhibitors and tend to exhibit high levels of NF-κB and AXL, while melanoma with high MITF levels exhibit low NF-κB activity and are more sensitive to MAPK inhibition [37,122,139]. AXL is not the main mediator of resistance in the MITF-low; NF-κB high cells, despite being able to confer resistance when overexpressed in MAPK-sensitive cell lines [137]. These MITF-low/NF-κB high cells may actually be regulated by high AP-1/TEAD transcription factor activity, which can confer resistance to MAPK inhibition [140].

Despite MITF high/NF-κB low melanoma showing sensitivity to MAPK inhibitors, the exact role of MITF in causing sensitivity to MAPK inhibitors is not clear as ectopic expression of MITF can also cause MAPK resistance [108,137] and NF-κB activity may be the main driver of resistance in melanoma naturally displaying the MITF low/NF-κB high phenotype [37]. If this is the case, treatment with Bromodomain and Extra Terminal inhibitors such as I-BET151 and JQ-1 potently suppress NF-κB signaling in melanoma, providing a rationale the use of these inhibitors in combination with MAPK inhibitors [54].

MITF activates the expression of anti-apoptotic genes BCL2A1, BCL2 and BIRC7 (MC-IAP/Livin) which may contribute to resistance that high MITF levels confer [65,136]. As MITF is normally suppressed by BRAF activity, a rebound in MITF levels following MAPK inhibition could be an early adaptive resistance.

#### 4.6.2. HDAC Inhibitors and MITF

MITF may be modulated by a number of epigenetic factors. HDAC inhibitors can suppress MITF expression [141] and HDAC inhibitors have been shown to blunt the effectiveness of ectopic MITF driven resistance to MAPK inhibitors [137]. However the activity of MITF may be increased by HDAC inhibitors as the suppressor of MITF transcription HINT1 associates with HDAC1 and mSIN3a to repress transcription of MITF targets [142]. MITF activity may also be modulated by heterochromatin protein 1, a protein associated with heterochromatin formation, although the direction of MITF regulation was not consistent between all cell lines [143].

#### 4.6.3. SWI/SNF and MITF

The SWI/SNF complex could be targeted to modulate MITF activity. The BRG1 containing SWI/SNF complex is required by MITF to activate melanocyte specific genes as MITF associates with SWI/SNF which increases chromatin accessibility at MITF target genes [59,144]. The SWI/SNF complex is also required to activate transcription of MITF itself, demonstrating the importance of the SWI/SNF complex in MITF processes [145]. BRG1 may be increased during melanoma progression [101,146], potentially causing a shift of MITF target genes towards anti-apoptotic signaling via BIRC7/ML-IAP, although conflicting reports exist about BRG1 expression in melanoma progression [147]. Another member of the SWI/SNF complex, SNF5 is also lost in melanoma progression and correlates to poor survival [148].

SWI/SNF complexes contain either BRG1 or BRM, which confer slightly different target specificity [59]. BRAF inhibits BRM expression and enhances BRG1 expression and following BRAF inhibition BRM is increased and BRG1 is reduced. BRAF inhibition leads to an increase in histone H4 acetylation at the BRM promoter (as did HDAC inhibitor treatment). HDAC3 and HDAC9 have been shown to regulate BRM expression—thus, linking HDAC with SWI/SNF [149]. It is likely that increases in BRM and an altered ratio of BRM:BRG1 expression following MAPK inhibition alters the transcriptional program of MITF leading to early alterations in drug sensitivity.

## 5. Overcoming Resistance to Immunotherapy

Melanoma cells have all the hallmarks of cells that should be eradicated by the immune system. They have a high mutation rate (the highest of all cancers [8]) and express a range of novel antigens, such as the cancer-testis-antigen family, which should provide plenty of antigens to attract the immune system. Tumor infiltrating lymphocytes are also frequently observed in melanoma tissue, indicating the presence of immune cells—and yet the immune system fails to kill the melanoma cells. This immune evasion is due to two main processes—inactivation of immune attack and resistance to apoptosis elicited by immune attack.

**Figure 3:**
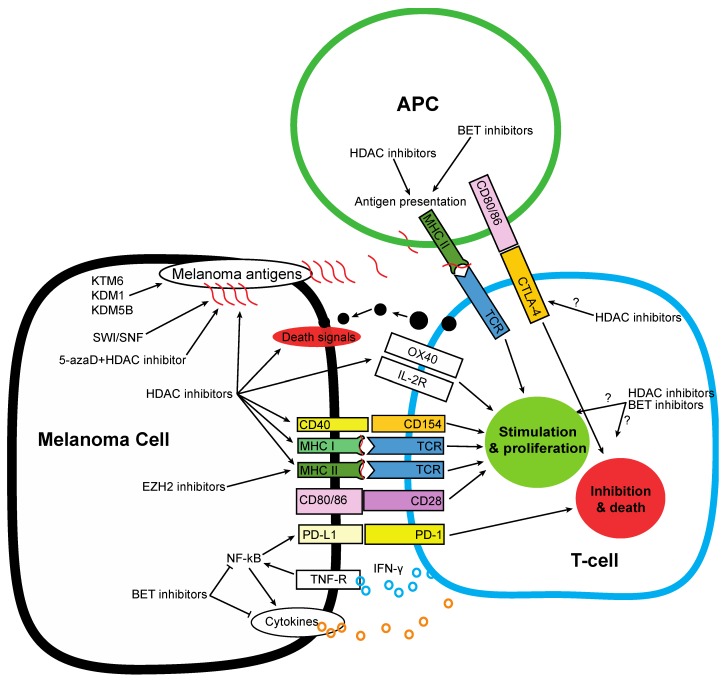
Melanoma cells evade immune attack using a number of mechanisms. Inhibitors of CTLA-4 and PD-1/PD-L1 have shown success in the clinic but some melanoma tumors are resistant. Epigenetic modulators can stimulate immune killing of melanoma by modulating antigen presentation, receptor expression and cytokine production in both melanoma and immune cells. However the effect of epigenetic modulators on immune cell viability and function is not fully understood and the effects differ for different immune cells. This makes the net result of many epigenetic modulators on anti-tumor immune function difficult to predict.

Immunotherapy against PD-1 and CTLA-4 targets two mechanisms that allow melanoma to evade immune attack—PD-L1 expression on melanoma cells to inactivate cytotoxic T-cells and CTLA-4 expression on lymphocytes which inhibits T-cell activation. The relatively high levels of primary resistance seen in approximately 30% of patients, as well as development of secondary resistance to these immune therapies in approximately 30%–40% of patients [150] is a major clinical problem and the causes of resistance are still being elucidated. Some resistance mechanisms are known and are driven by the biology of the melanoma, including downregulation of MHC molecules, loss of antigen expression, negative feedback causing PD-L1 upregulation in response to INF-γ production by T-cells and reduction in chemokine expression including CCL3, CSCL1, CXCL2 and CCL4 [151]. While these resistance mechanisms may be targeted by epigenetic modifiers (Figure 3), immune cells will also be affected by any drugs that are systemically administered and the effects may not always be advantageous.

### 5.1. Anti-Apoptotic Signaling Mediates Resistance to Immunotherapy

The general resistance of melanoma cells to apoptosis not only enables them to resist MAPK inhibitors, but also contributes to resistance to immunotherapy. Cells selected to be resistant to BRAF inhibition were also cross resistant to CTL and NK cell-death [152]. HDAC inhibitor restored sensitivity by shifting the balance of apoptotic protein towards apoptosis. This shows that histone modifiers can be used in the same way in immunotherapy as they might be for MAPK inhibitor therapy—to shift the balance towards a more pro-apoptotic state in the melanoma. A more direct extension of this is the increase in TRAIL sensitivity that HDAC inhibitors induce. TRAIL is used by T lymphocytes and NK cells to kill melanoma cells and TRAIL-resistance is another immune evasion mechanism [153]. Pan-HDAC inhibitors sensitize melanoma cells to TRAIL-induced death by down-regulating the inhibitory c-FLIP, increasing expression of TRAIL receptors such as DR4 and DR5 and shifting in expression of apoptotic proteins towards a state more permissive to apoptosis [92,94,99,154,155].

### 5.2. HDAC Inhibitors and Immune Function

While histone deacetylase inhibitors may drive melanoma toward an apoptotic state, they have a number of immune inhibitory effects [156]. HDAC inhibitors have been viewed as immunosuppressive, causing side effects such as lymphopenia, leukopenia, neutropenia, and thrombocytopenia, and are cytotoxic to PBMC’s at IC50 concentrations lower than melanoma cells, suggesting that they would negatively impact immunotherapy [157,158,159,160,161]. However, this view is no longer certain as HDAC inhibitors have a number of potentially beneficial effects and an increasing number of studies show HDAC inhibitors can improve immune related killing of melanoma.

HDAC inhibitors were shown to increase tumor immunogenicity as shown by studies on panobinostat [162]. Panobinostat enhanced the proliferation, retention and polyfunctional status of tumor specific T-cells in the B16 mouse model and induced higher IL-2R (CD25) and co-stimulatory OX-40 receptors in T-cells [163]. In these studies panobinostat improved the effectiveness of gp100 specific T-cell immunotherapy and maintained systemic pro-inflammatory levels. HDAC inhibitors may specifically enhance T-cell survival and function and can prevent activation-induced death of tumor infiltrating lymphocytes, promoting anti-tumor immunity [164]. These effects were predominantly mediated by CD4+ T-cells and enhanced memory T-cell populations. Activation of T-cells could also be targeted by HDAC inhibitors as the CTLA4 promoter is differentially acetylated in CD4+ *vs.* CD8+ T cells and may be able to be targeted to alter activation of these T-cell populations [165]. Cytokine production is also altered by HDAC inhibitors: IL-24, which activates monocytes and Th2 cells, is lost during melanoma progression but is re-expressed following HDAC inhibitor treatment [166].

HDAC inhibitors have also been used to enhance vaccine strategies. Depsipeptide promoted immune killing of B16/F10 melanoma cells [167] and only mice vaccinated with TSA treated B16 melanoma cells were effectively vaccinated from subsequent B16 tumor challenge [168,169]. In these studies, HDAC inhibitor treatment enhanced the expression of MHC class II, CD40 and B7-1/2 on B16 cells and vaccination with HDAC inhibitor-treated melanoma cells elicited tumor specific immunity in both prevention and treatment models. Cytotoxic and IFN-γ-producing cells were identified in splenocytes and CD4+, CD8+ T cells and NK cells were all involved in the induction of immunity. Apoptotic cells derived from HDAC inhibitor treatments, but not H_2_O_2_, significantly enhanced the effectiveness of the vaccine.

### 5.3. MHC Expression

Melanoma cells decrease MHC class I and II expression to evade immune response. The decrease of MHC class I expression in melanoma can be due to a transcriptional repression resulting from epigenetic modifications [170]. Histone deacetylases upregulate MHC class I expression in murine and human melanoma cells [170,171,172,173,174]. While class I HDAC are reported to be involved in MHC class I expression [174], the class IIb HDAC6 seems also involved as specific inhibition of HDAC6 via genetic means or with small molecule inhibitors Nexturastat A or Tubastatin A increased MHC class I expression in melanoma [175].

MHC class II, which present exogenous proteins following endocytosis can be downregulated in melanoma. HDAC treatment of B16 melanoma cells and human melanoma upregulated MHC class II expression and allowed them to become antigen presenting cells [169,174,176].

EZH2 causes H3K27me3 marks on the MHC2TA gene, leading to downregulation of MHC class II genes that may dampen the anti-tumor immune response [177,178]. Supporting this hypothesis, EZH2 has been implicated in the activation and maintenance of regulatory T-cells that suppress the immune system [179,180]. Histone methylation has also been reported to be an important determinant of cancer-testis antigen (NY-ESO-1, MAGEA1, MAGE-A3) expression in lung cancer, and repression of histone methyltransferase KMT6 and demethylases KDM1 and KDM5B induced antigen expression [181]. These antigens are commonly expressed in melanoma and may be similarly affected by histone methylation.

### 5.4. Expression and Presentation of Melanoma Antigens

A number of epigenetic modifiers have been shown to increase antigen exposure on melanoma. Antigen expression can be lost by melanoma, contributing to its immune evasion [182,183]. Depsipeptide, which preferentially inhibits class I HDAC, augmented NY-ESO-1 expression following 5-AzadC treatment (but not by itself) in melanoma and increased subsequent killing by CTL [184]. Likewise, pan-HDAC inhibitor TSA increased 5-aza-2′-deoxycytidine-induced expression of MAGE-A1 -A2, -A3 and -A12 genes. Examination of melanoma showed that MAGE-A is silenced by DNA hypermethylation and histone deacetylation [185]. Specific inhibition of class IIb HDAC6 can also increase tumor associated antigen production, such as gp100, Mart1 and Tryp1/2 in both human and murine melanoma cells [175]. Inhibition of HDAC11, the sole member of the HDAC IV family, can also increase antigen presentation by APCs [186]. Relatively little is known about HDAC11 but its expression is limited to relatively few tissues, making it an interesting target for immunotherapy. As well as increased antigen presentation, HDAC inhibitors may allow increased immune infiltration to the tumors as they increase ICAM-1 expression in tumor endothelial cells, increasing leucocyte interaction and infiltration [187].

### 5.5. SWI/SNF and Immunotherapy

SWI/SNF is also a potential target for immunomodulation. As SWI/SNF modulates melanocyte/melanoma specific genes, enhanced SWI/SNF may allow enhanced expression of melanoma specific antigens [144]. Melanomas undergoing the switch to a mesenchymal state are more able to escape T-cell immunity [188].

### 5.6. Histone Reader Proteins and Immunotherapy

Histone reader proteins may also have a role to play in immunotherapy, although like HDAC inhibitors, they also may negative impact on the immune system. BET inhibitor I-BET151 is a potent inhibitor of NF-κB activity and cytokine production in melanoma [54] and prevents the induction of PD-L1 expression on melanoma cells treated with IFN-γ [189]. The reduction in both PD-L1 and anti-inflammatory cytokine production could increase immune attack on melanoma. BET inhibitors may also have beneficial effects on immune function. BET inhibitor JQ1 increased inflammatory response of antigen presenting cells (APCs) by reducing expression PD-L1 and anti-inflammatory cytokines [190]. This lead to APC activation, increased priming of naïve CD4+ T-cells and restored responsiveness of tolerized CD4+ cells [190]. The positive effects of BET inhibition may still be offset by their adverse effects on immune function however. BET inhibitors suppress inflammation [191], macrophage cytokine production [192] and reduce dendritic cell (DC) maturation and function by inhibiting STAT5, thereby reducing DC-stimulation of CD4+ and CD8+ T-cell proliferation and function, leading to reduced T-cell response [193]. Early clinical use of BET inhibitor OTX015 show it is tolerated at clinically relevant doses, but can cause reversible neutropenia and thrombocytopenia, highlighting potentially negative effects on the immune system [194].

## 6. Future Directions

It is clear from the above review that treatment with targeted and immunotherapies involve a number of epigenetic changes that may be exploited to increase the effectiveness of these treatments in melanoma. In the case of targeted therapies the use of HDAC inhibitors alone or with BET protein inhibitors appears to be a promising approach to overcome a range of resistance mechanisms. Similarly preclinical studies on immunotherapy provide exciting opportunities to use inhibitors of HDACs, EZH2 and BET proteins to overcome resistance to immunotherapy induced by immune checkpoint inhibitors and to reverse resistance in ongoing treatments. Studies to build on the promising advances reviewed above are now needed.
